# Assessment of Suicidal Behaviors Among Individuals With Autism Spectrum Disorder in Denmark

**DOI:** 10.1001/jamanetworkopen.2020.33565

**Published:** 2021-01-12

**Authors:** Kairi Kõlves, Cecilie Fitzgerald, Merete Nordentoft, Stephen James Wood, Annette Erlangsen

**Affiliations:** 1 Australian Institute for Suicide Research and Prevention, Griffith University, Brisbane, Australia; 2 Danish Research Institute for Suicide Prevention, Mental Health Centre Copenhagen, Copenhagen, Denmark; 3 Department of Clinical Medicine, Faculty of Health Sciences, University of Copenhagen, Denmark; 4 Mental Health Centre Copenhagen, Copenhagen University Hospital, Copenhagen, Denmark; 5 Centre for Youth Mental Health, University of Melbourne, Melbourne, Australia; 6 Orygen, the National Centre of Excellence for Youth Mental Health, Parkville, Victoria, Australia; 7 School of Psychology, University of Birmingham, Edgbaston, United Kingdom; 8 Department of Mental Health, Johns Hopkins Bloomberg School of Public Health, Baltimore, Maryland; 9 Centre for Mental Health Research, Australian National University, Canberra, Australia

## Abstract

**Importance:**

There is limited evidence supporting an association of autism spectrum disorder (ASD) with suicidality and the risk factors for suicide attempt and suicide among people with ASD. Existing research highlights the need for national cohort studies.

**Objectives:**

To analyze whether people with ASD have higher rates of suicide attempt and suicide compared with people without ASD using national register data, identify potential risk factors for suicide attempt and suicide among those with ASD, and examine associations with comorbid disorders.

**Design, Setting, and Participants:**

In this cohort study, nationwide register data from January 1, 1995, to December 31, 2016, were gathered on 6 559 266 individuals in Denmark aged 10 years or older. Statistical analysis was performed from November 20, 2018, to November 21, 2020.

**Main Outcomes and Measures:**

Rates of suicide attempt and suicide among persons with ASD were compared with rates among persons without ASD, using Poisson regression models to calculate incidence rate ratios adjusted for sex, age, and time period.

**Results:**

Of the total study population of 6 559 266 individuals, 35 020 individuals (25 718 male [73.4%]; mean [SD] age at diagnosis, 13.4 [9.3] years) received a diagnosis of ASD. A total of 64 109 incidents of suicide attempts (587 [0.9%] among individuals with ASD) and 14 197 suicides (53 [0.4%] among individuals with ASD) were recorded. Persons with ASD had a more than 3-fold higher rate of suicide attempt (adjusted incidence rate ratio [aIRR], 3.19; 95% CI, 2.93-3.46) and suicide (aIRR, 3.75; 95% CI, 2.85-4.92) than those without ASD. For individuals with ASD, the aIRR for suicide attempt among female individuals was 4.41-fold (95% CI, 3.74-5.19) higher compared with male individuals; for individuals without ASD, the aIRR for female individuals was 1.41-fold (95% CI, 1.39-1.43) higher compared with male individuals. Higher rates of suicide attempt were noted across all age groups for those with ASD. Persons with a diagnosis of ASD only had an aIRR of 1.33 (95% CI, 0.99-1.78) for suicide attempt, whereas those with other comorbid disorders had an aIRR of 9.27 (95% CI, 8.51-10.10) for suicide attempt compared with those without any psychiatric disorders. A total of 542 of 587 individuals with ASD (92.3%) who attempted suicide had at least 1 other comorbid condition and 48 of 53 individuals with ASD (90.6%) who died by suicide had at least 1 other comorbid condition.

**Conclusions and Relevance:**

This nationwide retrospective cohort study found a higher rate of suicide attempt and suicide among persons with ASD. Psychiatric comorbidity was found to be a major risk factor, with more than 90% of those with ASD who attempted or died by suicide having another comorbid condition. Several risk factors are different from the risk factors in the general population, which suggests the need for tailored suicide prevention strategies.

## Key Points

**Question:**

Do people with autism spectrum disorder have higher rates of suicide attempt and suicide compared with people without autism spectrum disorder?

**Findings:**

In this nationwide retrospective cohort study that included 6 559 266 persons aged 10 years or older living in Denmark during the period from 1995 to 2016, individuals with a diagnosed autism spectrum disorder had more than 3-fold higher rates of suicide attempt and suicide compared with all other persons after adjusting for sex, age, and time period.

**Meaning:**

In this Danish cohort, diagnosis of an autism spectrum disorder was associated with suicide attempt and suicide.

## Teaser

This cohort study uses national register data to analyze whether people with ASD have higher rates of suicide attempt and suicide compared with people without ASD, identify potential risk factors for suicide attempts and suicide among those with ASD, and examine associations with comorbid disorders.

## Introduction

Autism spectrum disorder (ASD) comprises a set of chronic neurodevelopmental disorders with a wide range of symptoms and levels of severity.^1,2^ Globally, the prevalence of ASD has been estimated to be 1% to 1.5%,^3^ with a cumulative incidence up to 2.8% in recent birth cohorts in Denmark.^4^ Although the onset of ASD is generally in childhood, it may be recognized and diagnosed later in life.^2^ The number of children with a diagnosis of ASD has increased during recent decades,^1,2,5,6^ and professionals have debated whether this increase is due to changes in diagnostic criteria, increased clinical or parental awareness, or increased prevalence of etiologic factors.^2,4,5,6,7^

Lack of social integration, unemployment, and psychiatric disorders have been found to be associated with ASD in adults^8^; the same factors are associated with suicidal behavior,^9,10^ which would suggest a potential link between ASD and suicidal behavior. Nevertheless, little evidence from large-scale studies exists regarding an association between ASD and suicidality.^11,12,13,14,15^ A recent population-based case-cohort study from Sweden showed an increased risk of suicide and suicide attempt among those with ASD, especially among those without intellectual disability.^16^ It has yet to be determined what factors are associated with suicidal behavior in people with ASD and whether they differ from the factors associated with suicidal behavior in the population without ASD. To make evidence-based decisions and inform the design of intervention studies, there is a need for large-scale national cohort studies on the risk of suicide attempts and suicides among persons with ASD. Therefore, the aims of this retrospective cohort study were to analyze whether people with a diagnosis of ASD had higher rates of suicide attempts and suicides compared with people without ASD, identify risk factors for suicide attempt and suicide among those with ASD, and examine associations with psychiatric comorbid disorders.

## Methods

### Study Design and Population

A cohort design was applied to nationwide register data. The unique personal identification number assigned to each individual living in Denmark facilitated an individual-level data linkage of the Danish Civil Registration System^17^ with the Psychiatric Central Research Register (PCRR),^18^ the National Hospital Register,^19^ the Cause of Death Registry (CDR),^20^ the Populations Education Register, and the Income Statistics Register.^21^ From the latter 2 registers, data on educational level and socioeconomic status were gathered. The Civil Registration System contains information on sociodemographic characteristics, the PCRR and National Hospital Register contain information on morbidity, and the CDR contains information on mortality. This study, which used an anonymized, register-based data set, was approved by the Danish Data Protection Agency and followed the Strengthening the Reporting of Observational Studies in Epidemiology (STROBE) reporting guideline. Because this study is a national register–based study, all information is deidentified and is allowed to be linked for research purposes without need for consent.

All individuals living in Denmark from January 1, 1995, through December 31, 2016, were included in the cohort study. Given that suicide attempts and deaths are rare events among children, the inclusion age was set to 10 years or older.

### Outcomes

The primary outcome was suicide attempt, while death by suicide was examined separately as an exploratory outcome. Suicide attempt was recorded when an individual presented at either a psychiatric or somatic hospital or emergency department with one of the following *International Statistical Classification of Diseases and Related Health Problems, Tenth Revision* (*ICD-10*) codes: X60 to X84 or Y87.0, or when the reason for contact was listed as being self-harm. Death by suicide was identified in the CDR with the same *ICD-10* codes or when the manner of death was listed as suicide.^21^

### Exposures

#### ASD and Other Psychiatric Disorders

Information on ASD and other psychiatric diagnoses was obtained from the PCRR. This register contains data on inpatient admissions since 1969, while information on emergency department and outpatient contacts were available since 1995. During this period, diagnoses were recorded according to the *International Classification of Diseases, Eighth Revision* (*ICD-8*) and *ICD-10*. People who received the following codes for main diagnoses or subdiagnoses were considered as having ASD: *ICD-8* codes 299.00 to 299.01 and *ICD-10* codes F84.0 to F84.1 and F84.5 to F84.9.^7^ Dichotomization into low-functioning and high-functioning ASD was based on co-recorded *ICD* codes for intellectual disability (*ICD-8* codes 310-315 and *ICD-10* codes F70-F73 and F78-F79). We screened all supplementary diagnoses to capture comorbidity. Every person who had ever received a diagnosis of a mental disorder (see eTable 1 in the Supplement for complete list) was considered exposed from the date of diagnosis. In addition, parental psychiatric disorders and parental suicidal behavior (no parent vs ≥1 parent) were included.

#### Sociodemographic Factors and Physical Conditions

Several socidemographic factors were included as following: sex (male or female), age group (10-19, 20-29, 30-39, and ≥40 years), marital and cohabitational status (married, registered partnership, or cohabiting vs not), educational level (basic, vocational, high school, university degree, or unknown or missing), and socioeconomic status (employed, unemployed, disabled pensioner or retired, children or students, or unknown or missing). Age at ASD diagnosis was divided into 3 groups: children (<13 years), adolescent or young person (13-24 years), and adult (≥25 years). Presence of chronic physical disorders (0 [none] vs ≥1 [any]) was measured using the Charlson Comorbidity Index.^22^

### Follow-up

Follow-up began on January 1, 1995, and individuals reaching 10 years of age or migrating into the country were included on the date of those events. Persons were followed up until December 31, 2016, unless they migrated out of the country or died, in which case they were censored at the date of the respective event. In total, 233 493 persons emigrated and 1 148 407 died by causes other than suicide during the period from 1995 to 2016. Once the outcome of suicide attempt was observed, the individual was censored (ie, the follow-up ended). In addition, we did not consider the exposure (ie, ASD) if the person was reported with a first diagnosis of ASD and a suicide attempt on the same day; the suicide attempt was still counted but just as a suicide attempt among an unexposed individual.

### Statistical Analysis

Statistical analysis was performed from November 20, 2018, to November 21, 2020. Poisson regression models using person-years as offset were conducted using the PROC GENMOD procedure in SAS version 9.4 (SAS Institute Inc).^23^ The obtained estimates presented the suicide incidence rate, for instance, among people with a diagnosis of ASD compared with those with no disorders, expressed as incidence rate ratios. Covariates were added one at a time, while the model fit was evaluated by comparing the log likelihood of the models. We evaluated the model fit by comparing the log likelihood values of different models^24^ and checked that the model converged. We assessed for interactions between ASD and specific exposures. Multivariable models were adjusted for sex (male or female), age (10-19, 20-29, 30-39, or ≥40 years), and time period (1995-1999, 2000-2004, 2005-2009, or 2010-2016); the adjusted incidence rate ratios (aIRRs) with 95% CIs are presented. Considering that marital and cohabitation status, educational level, socioeconomic status, and other psychiatric disorders may be on the causal pathway between the ASD and suicidal behavior, they were not included as confounding factors. However, this issue was addressed in sensitivity analyses in which the main models for suicide and suicide attempt were adjusted for educational level, socioeconomic status, and marital and cohabitation status. Associations with other psychiatric disorders were examined in separate models. Additional sensitivity analyses were conducted in the study population, which was restricted to those born 1955 or later (ie, those who were ≤40 years in 1995). All variables were time varying and updated on either exact date of change or by calendar year (eg, change in the employment status).

## Results

Of the total study population of 6 559 266 persons aged 10 years or older living in Denmark during the period from 1995 to 2016, 35 020 individuals (25 718 male [73.4%]; mean [SD] age at diagnosis, 13.4 [9.3] years) received a diagnosis of ASD. eTable 2 in the Supplement provides a more detailed description of the people who received a diagnosis of ASD. A total of 64 109 people had at least 1 suicide attempt recorded, of whom 587 had ASD (0.9%) (Table 1). During the follow-up, 14 197 died by suicide; 53 of these individuals had ASD (0.4%).

**Table 1.** Incidence Rates per 100 000 and IRRs for Suicide Attempts

Characteristic	No. of suicide attempts	No. of person-years	Incidence rate per 100 000	IRR	aIRR (95% CI)^a^	aIRR (95% CI)^b^
ASD diagnosis						
ASD	587	219 985	266.84	4.21	3.19 (2.93-3.46)	3.19 (2.93-3.46)
No ASD	63 522	100 157 929	63.42	1 [Reference]	1 [Reference]	1 [Reference]
Sex^c^						
ASD						
Male	258	170 867	150.99	2.82	1.93 (1.71-2.18)	1 [Reference]
Female	329	49 118	669.82	12.49	8.51 (7.63-9.49)	4.41 (3.74-5.19)
No ASD						
Male	26 492	49 396 268	53.63	1 [Reference]	1 [Reference]	1 [Reference]
Female	37 030	50 761 660	72.95	1.36	1.41 (1.39-1.43)	1.41 (1.39-1.43)
Age group, y^d^						
ASD						
10-19	357	135 736	263.01	2.33	2.83 (2.55-3.15)	1 [Reference]
20-29	154	53 527	287.71	2.55	3.14 (2.68-3.68)	1.11 (0.92-1.34)
30-39	42	16 088	261.06	2.31	2.70 (1.99-3.65)	0.95 (0.69-1.31)
≥40	34	14 634	232.34	2.06	2.37 (1.69-3.32)	0.84 (0.59-1.19)
No ASD						
10-19	15 152	13 411 195	112.98	1 [Reference]	1 [Reference]	1 [Reference]
20-29	14 345	13 948 726	102.84	0.91	0.90 (0.88-0.92)	0.90 (0.88-0.92)
30-39	10 684	15 841 822	67.44	0.60	0.59 (0.57-0.60)	0.59 (0.57-0.60)
≥40	23 341	56 956 186	40.98	0.36	0.36 (0.35-0.37)	0.36 (0.35-0.37)
Age at first ASD diagnosis, y						
<13	194	135 145	143.55	2.26	1.82 (1.58-2.09)	1.82 (1.58-2.09)
13-24	334	67 544	494.49	7.80	4.61 (4.14-5.14)	4.61 (4.14-5.14)
≥25	59	17 295	341.14	5.38	6.78 (5.25-8.76)	6.78 (5.25-8.76)
No ASD	63 522	100 157 929	63.42	1 [Reference]	1 [Reference]	1 [Reference]
Educational level						
ASD						
Basic	442	130 179	339.53	3.01	2.36 (2.13-2.50)	1 [Reference]
Vocational or none	29	7494	386.95	3.43	3.02 (1.65-3.15)	1.28 (0.88-1.87)
High school	21	9117	230.33	2.04	1.49 (0.97-2.29)	0.63 (0.41-0.98)
University degree	11	4201	261.87	2.32	2.24 (1.37-3.45)	0.95 (0.52-1.73)
Missing or unknown	84	68 994	121.75	1.08	1.44 (1.53-2.21)	0.70 (0.55-0.88)
No ASD						
Basic	36 075	31 959 423	112.88	1 [Reference]	1 [Reference]	1 [Reference]
Vocational	12 864	29 160 589	44.11	0.39	0.43 (0.42-0.44)	0.43 (0.42-0.44)
High school	3594	6 985 781	51.45	0.46	0.30 (0.29-0.31)	0.30 (0.29-0.31)
University degree	4782	20 181 545	23.69	0.21	0.22 (0.21-0.22)	0.22 (0.21-0.22)
Missing or unknown	6207	11 870 590	52.29	0.46	0.64 (0.62-0.66)	0.64 (0.62-0.66)
Socioeconomic status						
ASD						
Employed	36	17 215	209.11	5.89	3.89 (2.81-5.40)	1 [Reference]
Unemployed	91	18 919	480.99	13.55	8.73 (7.10-10.73)	2.24 (1.52-3.30)
Disabled pensioner or retired	109	40 295	270.50	7.62	6.31 (5.23-7.62)	1.62 (1.11-2.36)
Child or student	266	120 594	220.58	6.21	4.38 (3.87-4.96)	1.13 (0.79-1.59)
Missing or unknown	85	22 961	370.19	10.43	6.31 (5.10-7.82)	1.62 (1.10-2.39)
No ASD						
Employed	18 778	52 892 345	35.50	1 [Reference]	1 [Reference]	1 [Reference]
Unemployed	9303	4 309 919	215.85	6.08	5.98 (5.83-6.13)	5.98 (5.83-6.13)
Disabled pensioner or retired	14 739	24 342 378	60.55	1.71	8.59 (8.37-8.83)	8.59 (8.37-8.83)
Child or student	11 957	11 854 203	100.87	2.84	1.54 (1.49-1.59)	1.54 (1.49-1.59)
Missing or unknown	8745	6 759 084	129.38	3.64	3.61 (3.51-3.71)	3.61 (3.51-3.71)
Relationship status						
ASD						
Married, registered partnership, or cohabiting	219	93 783	233.52	5.64	3.61 (3.16-4.13)	1 [Reference]
Not married, registered partnership, or cohabiting	351	108 980	322.08	7.78	5.62 (5.05-6.24)	1.56 (1.31-1.84)
Missing or unknown	17	17 221	98.72	2.38	3.28 (2.04-5.28)	0.91 (0.55-1.49)
No ASD						
Married, registered partnership, or cohabiting	26 824	64 794 545	41.40	1 [Reference]	1 [Reference]	1 [Reference]
Not married, registered partnership, or cohabiting	36 414	33 935 719	107.30	2.59	2.34 (2.30-2.37)	2.34 (2.30-2.37)
Missing or unknown	284	1 427 665	19.89	0.48	0.52 (0.46-0.58)	0.52 (0.46-0.58)
Charlson Comorbidity Index						
ASD						
None	518	197 384	262.43	4.28	3.31 (3.03-3.61)	1 [Reference]
≥1	69	22 601	305.30	4.97	4.00 (3.15-5.06)	1.21 (0.94-1.55)
No ASD						
None	52 230	85 106 307	61.37	1 [Reference]	1 [Reference]	1 [Reference]
≥1	11 292	15 051 622	75.02	1.22	2.13 (2.08-2.17)	2.13 (2.08-2.17)
Parental psychiatric disorders						
ASD						
None	405	162 820	248.74	4.55	3.50 (3.17-3.86)	1 [Reference]
≥1 Parent	182	57 165	318.38	5.83	4.46 (3.86-5.16)	1.28 (1.07-1.52)
No ASD						
None	49 574	90 768 960	54.62	1 [Reference]	1 [Reference]	1 [Reference]
≥1 Parent	13 948	9 388 969	148.56	2.72	2.26 (2.21-2.30)	2.26 (2.21-2.30)
Parental suicidal behavior						
ASD						
None	531	207 291	256.16	4.31	3.24 (2.97-3.53)	1 [Reference]
≥1 Parent	56	12 694	441.15	7.42	5.53 (4.25-7.19)	1.71 (1.30-2.25)
No ASD						
None	58 031	97 633 520	59.44	1 [Reference]	1 [Reference]	1 [Reference]
≥1 Parent	5491	2 524 408	217.52	3.66	2.93 (2.85-3.02)	2.93 (2.85-3.02)

Abbreviations: aIRR, adjusted incidence rate ratio; ASD, autism spectrum disorder; IRR, incidence rate ratio.

^a^ Shared analysis; adjusted for age, sex, and period.

^b^ Separate analysis for those with and without ASD; adjusted for age, sex, and period.

^c^ Adjusted for age and period.

^d^ Adjusted for sex and period.

### Suicide Attempt

The incidence rates of suicide attempt were 266.8 per 100 000 person-years among those with ASD and 63.4 per 100 000 person-years among those without ASD (Table 1). When adjusting for sex, age, and period, we found that persons with ASD had a higher rate of suicide attempt (aIRR, 3.19; 95% CI, 2.93-3.46) compared with those without ASD.

Analysis by sex showed that male individuals with ASD had a 1.93-fold (95% CI, 1.71-2.18) higher incidence rate of suicide attempt compared with male individuals without ASD (Table 1). For individuals with ASD, the aIRR for female individuals was 4.41-fold (95% CI, 3.74-5.19) higher compared with male individuals; for individuals without ASD, the aIRR for female individuals was 1.41-fold (95% CI, 1.39-1.43) higher compared with male individuals. With the use of persons aged 10 to 19 years as a reference group, a different pattern for those with ASD than for the general population was revealed; persons with ASD did not show differences between age groups. However, for those without ASD, the aIRR decreased with age. The rate ratio for suicide attempt increased with the age at first diagnosis.

People with ASD who were unemployed had a 2.24-fold (95% CI, 1.52-3.30) higher incidence rate of suicide attempt compared with those who were employed; for individuals without ASD, those who were unemployed had a 5.89-fold (95% CI, 5.83-6.13) higher incidence rate (Table 1). The difference between those with and those without ASD was even wider for disabled pensioners and retired individuals; they had a 1.62-fold (95% CI, 1.11-2.36) higher rate compared with employed individuals among those with ASD and an 8.59-fold (95% CI, 8.37-8.83) higher rate compared with employed individuals among those without ASD. However, compared with the respective socioeconomic groups, the rate of suicide attempt was highest for employed individuals: 3.89-fold (95% CI, 2.81-5.40) higher for those with ASD compared with those without ASD. For people with ASD, a higher rate of suicide attempt was noted across all educational categories compared with those without ASD. More specifically, for those without ASD, the rate of suicide attempt decreased with educational level, but among those with ASD, individuals with vocational and university education did not have a lower rate of suicide attempt compared with individuals with a basic education. Individuals with ASD who were in a relationship (married, civil union, or cohabiting) had a 3.61-fold (95% CI, 3.16-4.13) higher rate compared with those without ASD. For individuals with ASD, the aIRR for those not in a relationship was 1.56-fold (95% CI, 1.31-1.84) higher compared with those in a relationship; for individuals without ASD, the aIRR for those not in a relationship was 2.34-fold (95% CI, 2.30-2.37) higher compared with those in a relationship. Physical comorbidities were associated with a 2.13-fold (95% CI, 2.08-2.17) higher rate of suicide attempt for those without ASD vs no change for those with ASD (aIRR, 1.21; 95% CI, 0.94-1.55).

A total of 72.5% of those with ASD (25 401 of 35 020) had received a diagnosis of other psychiatric disorders (eTable 2 in the Supplement). The aIRR for individuals with a diagnosis only of ASD was 1.33 (95% CI, 0.99-1.78), while those with other comorbid disorders had an aIRR of 9.27 (95% CI, 8.51-10.10) compared with individuals without any psychiatric disorders (Table 2). The aIRR was higher for persons with psychiatric disorders other than ASD (aIRR, 21.00; 95% CI, 20.67-21.34). The most prevalent type of psychiatric comorbidity among those with ASD was attention-deficit/hyperactivity disorder (ADHD); 11 456 [32.7%]), followed by those with anxiety, dissociative, stress-related, and somatoform disorders (9646 [27.5%]) and affective disorders (5770 [16.5%]) (eTable 2 in the Supplement). Although higher suicide attempt rates were noted for most of the examined comorbid psychiatric disorders, no difference was noted for ADHD and intellectual disability. Adjusted incidence rate ratios were highest for relatively rare comorbidities, including posttraumatic stress disorder, substance use disorders, and borderline personality disorder (Table 2).

**Table 2.** IRRs for Suicide Attempts by ASD and Other Psychiatric Comorbidity

Characteristic	No. of suicide attempts	No. of person-years	Incidence rate per 100 000	IRR	aIRR (95% CI)^a^
Any psychiatric disorder					
No psychiatric disorder	27 799	93 377 383	29.77	1 [Reference]	1 [Reference]
ASD	45	76 450	58.86	1.98	1.33 (0.99-1.78)
Any other psychiatric disorder	35 723	6 780 546	526.85	17.70	21.00 (20.67-21.34)
ASD and any comorbid disorder	542	143 535	377.61	12.68	9.27 (8.51-10.10)
Substance use disorder					
No ASD and no substance use disorder	51 213	98 732 586	51.87	1 [Reference]	1 [Reference]
ASD	515	215 041	239.49	4.62	3.17 (2.91-3.46)
Substance use disorder	12 309	1 425 343	863.58	16.65	25.13 (24.62-25.66)
ASD and substance use disorder	72	4944	1456.25	28.07	25.62 (20.33-32.29)
Schizophrenia^b^					
No ASD and no schizophrenia	60 294	99 639 079	60.51	1 [Reference]	1 [Reference]
ASD	509	210 391	241.93	4.00	2.91 (2.67-3.18)
Schizophrenia	3228	518 850	622.15	10.28	12.13 (11.70-12.57)
ASD and schizophrenia	78	9594	812.99	13.44	11.95 (9.56-14.92)
SSD					
No ASD and no SSD	56 991	99 015 403	57.56	1 [Reference]	1 [Reference]
ASD	421	197 430	213.24	3.70	2.64 (2.40-2.91)
SSD	6531	1 142 525	571.63	9.93	11.95 (11.65-12.27)
ASD and SSD	166	22 555	735.99	12.79	10.02 (8.60-11.67)
Affective disorders					
No ASD and no affective disorders	48 948	97 782 484	50.06	1 [Reference]	1 [Reference]
ASD	356	196 397	181.27	3.62	2.54 (2.29-2.82)
Affective disorders	14 574	2 375 445	613.53	12.26	16.54 (16.22-16.87)
ASD and affective disorders	231	23 588	979.31	19.56	13.36 (11.74-15.22)
Depression^c^					
No ASD and no depression	50 313	98 065 774	51.31	1 [Reference]	1 [Reference]
ASD	371	199 353	186.10	3.63	2.58 (2.33-2.86)
Depression	13 209	2 092 155	631.36	12.31	16.42 (16.09-16.75)
ASD and depression	216	20 632	1046.90	20.41	13.78 (12.05-15.76)
Bipolar disorder^c^					
No ASD and no bipolar disorder	61 582	99 822 858	61.69	1 [Reference]	1 [Reference]
ASD	566	217 799	259.87	4.21	3.08 (2.83-3.35)
Bipolar disorder	1940	335 070	578.98	9.39	12.98 (12.40-13.58)
ASD and bipolar disorder	21	2186	960.61	15.57	12.72 (8.30-19.52)
ADSO					
No ASD and no ADSO	42 061	97 005 033	43.36	1 [Reference]	1 [Reference]
ASD	242	182 021	132.95	3.07	2.18 (1.92-2.47)
ADSO	21 461	3 152 896	680.68	15.70	18.37 (18.06-18.69)
ASD and ADSO	345	37 964	908.76	20.96	14.80 (13.30-16.46)
Anxiety^d^					
No ASD and no anxiety	59 796	99 242 157	60.25	1 [Reference]	1 [Reference]
ASD	514	208 138	246.95	4.10	3.02 (2.76-3.29)
Anxiety	3726	915 771	406.87	6.75	7.55 (7.30-7.80)
ASD and anxiety	73	11 847	616.19	10.23	7.02 (5.58-8.84)
OCD^d^					
No ASD and no OCD	62 978	100 013 597	62.97	1 [Reference]	1 [Reference]
ASD	544	144 332	376.91	5.99	3.05 (2.80-3.33)
OCD	539	210 642	255.88	4.06	4.71 (4.33-5.13)
ASD and OCD	48	9342	513.79	8.16	5.52 (4.15-7.32)
PTSD^d^					
No ASD and no PTSD	62 719	100 001 653	62.72	1 [Reference]	1 [Reference]
ASD	573	219 452	261.10	4.16	3.10 (2.85-3.37)
PTSD	803	156 275	513.84	8.19	8.96 (8.35-9.61)
ASD and PTSD	14	533	2627.36	41.89	33.39 (19.77-56.39)
Eating disorders					
No ASD and no eating disorders	62 089	99 935 650	62.13	1 [Reference]	1 [Reference]
ASD	538	216 835	248.12	3.99	3.00 (2.75-3.26)
Eating disorders	1433	222 279	644.69	10.38	6.60 (6.26-6.96)
ASD and eating disorders	49	3150	1555.43	25.04	13.89 (10.50-18.39)
Personality disorders					
No ASD and no personality disorder	48 897	96 900 965	50.46	1 [Reference]	1 [Reference]
ASD	455	189 889	239.61	4.75	2.90 (2.64-3.18)
Personality disorder(s)	14 625	3 256 964	449.04	8.90	12.58 (12.34-12.83)
ASD and personality disorder(s)	132	30 096	438.59	8.69	7.02 (5.92-8.33)
Bordeline personality disorder					
No ASD and no borderline personality disorder	61 172	99 994 448	61.18	1 [Reference]	1 [Reference]
ASD	559	218 333	256.03	4.19	3.07 (2.83-3.34)
Borderline personality disorder	2350	163 481	1437.48	23.50	20.08 (19.26-20.94)
ASD and borderline personality disorder	28	1651	1695.52	27.72	21.53 (14.86-31.19)
ADHD					
No ASD and no ADHD	62 097	99 869 997	62.18	1 [Reference]	1 [Reference]
ASD	428	165 503	258.61	4.16	3.17 (2.88-3.48)
ADHD	1425	287 931	494.91	7.96	6.21 (5.89-6.55)
ASD and ADHD	159	54 482	291.84	4.69	3.83 (3.28-4.48)
ODD or CD^e^					
No ASD and no ODD or CD	62 430	99 996 521	62.43	1 [Reference]	1 [Reference]
ASD	510	205 600	248.05	3.97	3.02 (2.76-3.30)
ODD or CD	1092	161 407	676.55	10.84	7.70 (7.25-8.18)
ASD and ODD or CD	77	14 385	535.30	8.57	6.23 (4.98-7.79)
Intellectual disability					
No ASD and no intellectual disability	62 793	99 846 868	62.89	1 [Reference]	1 [Reference]
ASD	526	183 913	286.01	4.55	3.13 (2.91-3.37)
Intellectual disability	729	311 061	234.36	3.73	3.38 (3.10-3.68)
ASD and intellectual disability	61	36 072	169.10	2.69	2.08 (1.62-2.68)

Abbreviations: ADHD, attention-deficit/hyperactivity disorder; ADSO, anxiety, dissociative, stress-related, and somatoform disorders; aIRR, adjusted incidence rate ratio; ASD, autism spectrum disorder; CD, conduct disorder; IRR, incidence rate ratio; OCD, obsessive-compulsive disorder; ODD, oppositional defiant disorder; PTSD, posttraumatic stress disorder; SSD, schizophrenia spectrum disorder.

^a^ Adjusted for age, sex, and period.

^b^ Subgroup of SSD.

^c^ Subgroup of affective disorders.

^d^ Subgroup of ADSO and other nonpsychotic mental disorders.

^e^ Subgroup of neurodevelopmental disorders.

People with a parent who had a psychiatric disorder had a 2.26-fold (95% CI, 2.21-2.30) higher rate of suicide attempt if they did not have ASD and a 1.28-fold (95% CI, 1.07-1.52) higher rate if they did have ASD. Relatively similar trends were measured for parental suicidal behavior (Table 1).

### Suicide

With respect to suicide, persons with ASD had a 3.75-fold (95% CI, 2.85-4.92) higher rate compared with those without ASD after adjusting for sex, age, and period (Table 3). Male individuals with ASD had an aIRR of 3.48 (95% CI, 2.57-4.74), and female individuals with ASD had an aIRR of 2.63 (95% CI, 1.46-4.76), compared with those without ASD. For individuals with ASD, there was no difference by sex in suicide rate (aIRR, 0.75; 95% CI, 0.39-1.46); for those without ASD, female individuals had a 0.36-fold (95% CI, 0.35-0.37) lower suicide rate compared with male individuals. The aIRR increased gradually with age for those without ASD; however, this pattern was not confirmed for those with ASD. Although most results showed relatively similar trends to suicide attempts, they were less pronounced and included some differences. For example, in people without ASD, those who were not married or cohabiting had a 2.81-fold (95% CI, 2.72-2.91) higher rate compared with those who were married or cohabiting; people with ASD who were married or cohabiting had no difference in the suicide rate than those who were not married or cohabiting (0.77; 95% CI, 0.42-1.38). With regard to psychiatric comorbidities, similarly, 48 of 53 individuals (90.6%) had at least 1 comorbid condition; the most prevalent type of psychiatric comorbidity among those with ASD was affective disorders (27 of 53 [50.9%]), followed by anxiety, dissociative, stress-related, and somatoform disorders (25 of 53 [47.2%]) and schizophrenia spectrum disorders (24 of 53 [45.3%]). The IRRs for suicide were highest for the comorbidity with depression and substance use disorders (Table 4).

**Table 3.** Incidence Rates per 100 000 and IRRs for Suicide

Characteristic	No. of suicides	No. of person-years	Incidence rate per 100 000	IRR	aIRR (95% CI)^a^	aIRR (95% CI)^b^
ASD diagnosis						
ASD	53	225 076	23.55	1.68	3.75 (2.85-4.92)	3.75 (2.85-4.92)
No ASD	14 144	100 749 269	14.04	1 [Reference]	1 [Reference]	1 [Reference]
Sex^c^						
ASD						
Male	42	173 296	24.24	1.18	3.48 (2.57-4.74)	1 [Reference]
Female	11	51 780	21.24	1.03	2.63 (1.46-4.76)	0.75 (0.39-1.46)
No ASD						
Male	10 227	49 640 038	20.60	1 [Reference]	1 [Reference]	1 [Reference]
Female	3917	51 109 232	7.66	0.37	0.36 (0.35-0.37)	0.36 (0.35-0.37)
Age group, y^d^						
ASD						
10-19	16	137 108	11.67	4.79	4.27 (2.59-7.06)	1 [Reference]
20-29	23	55 755	41.25	16.92	15.48 (10.14-23.63)	3.62 (1.91-6.86)
30-39	8	17 028	46.98	19.27	17.38 (8.62-35.05)	4.07 (1.74-9.50)
≥40	6	15 184	39.52	16.21	15.42 (6.88-34.58)	3.61 (1.41-9.22)
No ASD						
10-19	328	13 451 226	2.44	1 [Reference]	1 [Reference]	1 [Reference]
20-29	1194	14 098 556	8.47	3.47	3.42 (3.03-3.86)	3.42 (3.03-3.86)
30-39	1912	15 974 367	11.97	4.91	4.82 (4.29-5.42)	4.82 (4.29-5.42)
≥40	10 710	57 225 120	18.72	7.68	7.88 (7.06-8.80)	7.88 (7.06-8.80)
Age at first ASD diagnosis, y						
<13	10	136 191	7.34	0.52	1.79 (0.96-3.33)	1.79 (0.96-3.33)
13-24	32	70 625	45.31	3.23	6.21 (4.38-8.80)	6.21 (4.38-8.80)
≥25	11	18 261	60.24	4.29	3.93 (2.18-7.10)	3.93 (2.18-7.10)
No ASD	14 144	100 749 269	14.04	1 [Reference]	1 [Reference]	1 [Reference]
Educational level						
ASD						
Basic	37	133 916	27.63	1.61	3.14 (2.27-4.35)	1 [Reference]
Vocational or none	<4	7859	NA	NA	2.25 (0.73-6.99)	0.72 (0.22-2.33)
High school	4	9485	42.17	2.46	3.28 (1.23-8.76)	1.05 (0.37-2.93)
University degree	4	4384	91.23	5.32	5.07 (1.90-13.53)	1.61 (0.58-4.53)
Missing or unknown	5	69 432	7.20	0.42	2.56 (1.06-6.16)	0.81 (0.32-2.07)
No ASD						
Basic	5533	32 276 141	17.14	1 [Reference]	1 [Reference]	1 [Reference]
Vocational or none	4386	29 309 989	14.96	0.87	0.68 (0.65-0.70)	0.68 (0.65-0.70)
High school	684	7 027 781	9.73	0.57	0.70 (0.65-0.76)	0.70 (0.65-0.76)
University degree	2220	20 241 447	10.97	0.64	0.55 (0.53-0.58)	0.55 (0.53-0.58)
Missing or unknown	1321	11 893 911	11.11	0.65	1.08 (1.02-1.15)	1.08 (1.02-1.15)
Socioeconomic status						
ASD						
Employed	<4	17 685	NA	NA	2.09 (0.52-8.36)	1 [Reference]
Unemployed	7	20 074	34.87	4.81	7.84 (3.73-16.49)	3.76 (0.78-18.08)
Disabled pensioner or retired	18	42 144	42.71	5.89	6.59 (4.15-10.48)	3.16 (0.73-13.60)
Child or student	9	121 698	7.40	1.02	5.31 (2.74-10.29)	2.54 (0.55-11.80)
Missing or unknown	17	23 475	72.42	9.99	31.73 (19.62-51.33)	15.19 (3.51-65.80)
No ASD						
Employed	3847	53 089 677	7.25	1 [Reference]	1 [Reference]	1 [Reference]
Unemployed	817	4 424 475	18.47	2.55	2.87 (2.66-3.10)	2.87 (2.66-3.10)
Disabled pensioner or retired	6164	24 535 546	25.12	3.47	5.27 (5.00-5.56)	5.27 (5.00-5.56)
Child or student	299	11 898 272	2.51	0.35	1.45 (1.27-1.67)	1.45 (1.27-1.67)
Missing or unknown	3017	6 801 298	44.36	6.12	11.98 (11.41-12.58)	11.98 (11.41-12.58)
Relationship status						
ASD						
Married, registered partnership, or cohabiting	17	112 706	15.08	1.65	7.50 (4.64-12.12)	1 [Reference]
Not married, registered partnership, or cohabiting	32	95 128	33.64	3.67	5.74 (4.05-8.13)	0.77 (0.42-1.38)
Missing or unknown	4	17 242	23.20	2.53	55.45 (19.78-155.41)	7.40 (2.39-22.88)
No ASD						
Married, registered partnership, or cohabiting	5900	64 345 501	9.17	1 [Reference]	1 [Reference]	1 [Reference]
Not married, registered partnership, or cohabiting	8193	34 976 078	23.42	2.55	2.81 (2.72-2.91)	2.81 (2.72-2.91)
Missing or unknown	51	1 427 691	3.57	0.39	0.84 (0.63-1.10)	0.84 (0.63-1.10)
Charlson Comorbidity Index						
ASD						
None	51	201 765	25.28	2.12	4.53 (3.43-5.98)	1 [Reference]
≥1	<4	23 311	NA	NA	1.46 (0.37-5.85)	0.32 (0.08-1.33)
No ASD						
None	10 203	85 568 206	11.92	1 [Reference]	1 [Reference]	1 [Reference]
≥1	3941	15 181 063	25.96	2.18	1.80 (1.73-1.87)	1.80 (1.73-1.87)
Parental psychiatric disorders						
ASD						
None	39	166 188	23.47	1.74	4.50 (3.28-6.18)	1 [Reference]
≥1 Parent	14	58 888	23.77	1.76	4.56 (2.69-7.71)	1.01 (0.55-1.86)
No ASD						
None	12 295	91 213 021	13.48	1 [Reference]	1 [Reference]	1 [Reference]
≥1 Parent	1849	9 536 248	19.39	1.44	2.00 (1.90-2.10)	2.00 (1.90-2.10)
Parental suicidal behavior						
ASD						
None	47	211 893	22.18	1.62	3.97 (2.97-5.30)	1 [Reference]
≥1 Parent	6	13 183	45.51	3.33	7.61 (3.42-16.96)	1.92 (0.82-4.49)
No ASD						
None	13 435	98 166 692	13.69	1 [Reference]	1 [Reference]	1 [Reference]
≥1 Parent	709	2 582 577	27.45	2.01	2.70 (2.50-2.92)	2.70 (2.50-2.92)

Abbreviations: ASD, autism spectrum disorder; IRR, incidence rate ratio.

^a^ Shared analysis; adjusted for age, sex, and period.

^b^ Separate analysis for those with and without ASD; adjusted for age, sex, and period.

^c^ Adjusted for age and period.

^d^ Adjusted for sex and period.

**Table 4.** IRRs for Suicide Deaths by ASD and Other Psychiatric Comorbidity

Characteristic	No. of suicides	No. of person-years	Incidence rate per 100 000	IRR	aIRR (95% CI)^a^
Any psychiatric disorder					
No psychiatric disorder	7439	93 538 565	7.95	1 [Reference]	1 [Reference]
ASD	5	76 654	6.52	0.82	2.26 (0.94-5.44)
Any other psychiatric disorder	6705	7 210 705	92.99	11.69	12.27 (11.87-12.69)
ASD and any comorbid disorder	48	148 423	32.34	4.07	8.47 (6.37-11.28)
Substance use disorder					
No ASD and no substance use disorder	11 568	99 162 774	11.67	1 [Reference]	1 [Reference]
ASD	41	219 130	18.71	1.60	3.63 (2.66-4.94)
Substance use disorder	2576	1 586 496	162.37	13.92	10.82 (10.36-11.29)
ASD and substance use disorder	12	5946	201.81	17.30	19.44 (11.03-34.26)
Schizophrenia^b^					
No ASD and no schizophrenia	13 200	100 178 925	13.18	1 [Reference]	1 [Reference]
ASD	42	214 246	19.60	1.49	3.66 (2.69-4.96)
Schizophrenia	944	570 344	165.51	12.56	11.06 (10.35-11.82)
ASD and schizophrenia	11	10 830	101.57	7.71	8.75 (4.84-15.81)
SSD					
No ASD and no SSD	12 349	99 514 993	12.41	1 [Reference]	1 [Reference]
ASD	29	200 469	14.47	1.17	2.91 (2.02-4.20)
SSD	1795	1 234 277	145.43	11.72	10.75 (10.23-11.30)
ASD and SSD	24	24 608	97.53	7.86	10.74 (7.05-15.73)
Affective disorders					
No ASD and no affective disorders	10 623	98 179 747	10.82	1 [Reference]	1 [Reference]
ASD	26	199 119	13.06	1.21	2.69 (1.83-3.96)
Affective disorders	3521	2 569 522	137.03	12.66	13.55 (13.03-14.09)
ASD and affective disorders	27	25 957	104.02	9.61	17.60 (12.05-25.71)
Depression^c^					
No ASD and no depression	10 992	98 479 370	11.16	1 [Reference]	1 [Reference]
ASD	28	202 243	13.84	1.24	2.78 (1.91-4.04)
Depression	3152	2 269 899	138.86	12.44	13.59 (13.05-14.15)
ASD and depression	25	22 833	109.49	9.81	19.04 (12.84-28.23)
Bipolar disorder^c^					
No ASD and no bipolar disorder	13 443	100 384 641	13.39	1 [Reference]	1 [Reference]
ASD	49	222 671	22.01	1.64	3.78 (2.85-5.02)
Bipolar disorder	701	364 628	192.25	14.36	13.30 (12.32-14.35)
ASD and bipolar disorder	4	2405	166.34	12.42	16.17 (6.07-43.11)
ADSO					
No ASD and no ADSO	10 897	97 306 366	11.20	1 [Reference]	1 [Reference]
ASD	28	183 875	15.23	1.36	3.23 (2.22-4.68)
ADSO	3247	3 442 903	94.31	8.42	9.79 (9.41-10.19)
ASD and ADSO	25	41 201	60.68	5.42	12.30 (8.29-18.24)
Anxiety^d^					
No ASD and no anxiety	13 474	99 781 691	13.50	1 [Reference]	1 [Reference]
ASD	49	212 442	23.07	1.71	3.99 (3.01-5.29)
Anxiety	670	967 578	69.25	5.13	6.05 (5.60-6.55)
ASD and anxiety	4	12 635	31.66	2.34	5.14 (1.93-13.71)
OCD^d^					
No ASD and no OCD	14 066	100 597 040	13.98	1 [Reference]	1 [Reference]
ASD	52	215 430	24.14	1.73	4.05 (3.08-5.34)
OCD	78	152 229	51.24	3.66	5.83 (4.66-7.28)
ASD and OCD	<4	9646	NA	NA	1.82 (0.26-12.92)
PTSD^d^					
No ASD and no PTSD	14 028	100 579 757	13.95	1 [Reference]	Model did not converge
ASD	53	224 416	23.62	1.69	NA
PTSD	116	169 512	68.43	4.91	NA
ASD and PTSD	<4	660	NA	NA	NA
Eating disorders					
No ASD and no eating disorders	14 039	100 508 092	13.97	1 [Reference]	Model did not converge
ASD	53	221 484	23.93	1.71	NA
Eating disorders	105	241 177	43.54	3.12	NA
ASD and eating disorders	<4	3592	NA	NA	NA
Personality disorder(s)					
No ASD and no personality disorder(s)	10 886	97 296 210	11.19	1 [Reference]	1 [Reference]
ASD	35	192 896	18.14	1.62	3.94 (2.81-5.51)
Personality disorder(s)	3258	3 453 060	94.35	8.43	7.54 (7.24-7.84)
ASD and personality disorder(s)	18	32 180	55.94	5.00	5.24 (3.30-8.32)
Borderline personality disorder					
No ASD and no borderline personality disorder	13 811	100 538 377	13.74	1 [Reference]	1 [Reference]
ASD	51	222 804	22.89	1.67	3.94 (2.98-5.20)
Borderline personality disorder	333	210 892	157.90	11.49	17.64 (15.81-19.69)
ASD and borderline personality disorder	<4	2272	NA	NA	10.36 (2.59-41.42)
ADHD					
No ASD and no ADHD	14 054	100 443 078	13.99	1 [Reference]	1 [Reference]
ASD	39	169 425	23.02	1.65	3.58 (2.61-4.92)
ADHD	90	306 191	29.39	2.10	4.64 (3.76-5.72)
ASD and ADHD	14	55 651	25.16	1.80	6.18 (3.65-10.47)
ODD or CD^e^					
No ASD and no ODD or CD	14 068	100 574 905	13.99	1 [Reference]	1 [Reference]
ASD	48	209 971	22.86	1.63	3.86 (2.90-5.14)
ODD or CD	76	174 364	43.59	3.12	5.24 (4.18-6.58)
ASD and ODD or CD	5	15 105	33.10	2.37	5.78 (2.40-13.91)
Intellectual disability					
No ASD and no intellectual disability	14 081	100 428 942	14.02	1 [Reference]	1 [Reference]
ASD	52	188 320	27.61	1.97	4.73 (3.59-6.22)
Intellectual disability	63	320 327	19.67	1.40	1.75 (1.37-2.25)
ASD and intellectual disability	<4	36 756	NA	NA	0.41 (0.06-2.89)

Abbreviations: ADHD, attention-deficit/hyperactivity disorder; ADSO, anxiety, dissociative, stress-related, and somatoform disorders; aIRR, adjusted incidence rate ratio; ASD, autism spectrum disorder; CD, conduct disorder; IRR, incidence rate ratio; NA, not applicable; OCD, obsessive-compulsive disorder; ODD, oppositional defiant disorder; PTSD, posttraumatic stress disorder; SSD, schizophrenia spectrum disorder.

^a^ Adjusted for age, sex, and period.

^b^ Subgroup of SSD.

^c^ Subgroup of affective disorders.

^d^ Subgroup of ADSO and other nonpsychotic mental disorders.

^e^ Subgroup of neurodevelopmental disorders.

### Sensitivity Analyses

A significant association remained between ASD suicide attempt (aIRR, 1.97; 95% CI, 1.81-2.14) and suicide (aIRR, 2.10; 95% CI, 1.81-2.14) when additionally adjusting for educational level, socioeconomic status, and marital and cohabitational status. Also, when we restricted the analyses to those born after 1955, persons with ASD were found to have an aIRR of 3.18 (95% CI, 2.92-3.45) for suicide attempt and an aIRR of 3.83 (95% CI, 2.91-5.05) for suicide compared with those without ASD.

## Discussion

This registry-based national cohort study showed that persons with a diagnosis of ASD have more than 3-fold higher rates of suicide attempt and suicide compared with those without ASD, after adjusting for sex, age, and time period. Factors that have been identified as protective against suicide attempt in the general population, such as older age and higher educational level, were not found to have this association among individuals with ASD, and some factors, such as being married or cohabiting and employed, were associated with being less protective among those with ASD. Most factors associated with suicide in the general population were not associated with suicide among those with ASD (eg, male sex or not being married or cohabiting). Psychiatric comorbidity was found to be a major risk factor, with more than 90% of those with ASD who attempted or died by suicide having another comorbid condition (with anxiety and affective disorders being most common). These factors are crucial for assessing suicide risk by practitioners working with people with ASD.

To our knowledge, this is the first nationwide cohort study to examine the association of ASD with suicide attempt as well as with suicide. A recent prospective birth cohort study from the United Kingdom^25^ showed an association of the impairment of social communication with suicidal thoughts, suicidal plans, and self-harm in adolescence, but it was unable to show an association between ASD and suicidality (ideation and self-harm) owing to limited statistical power. A nested case-control study from Taiwan^26^ found that ASD was significantly associated with suicide attempts among adolescents (12-17 years) and young adults (18-29 years) during the follow-up period, showing a similar risk for both age groups. It has been suggested that adolescents and young adults with ASD have a higher risk of suicide attempts^27^; nevertheless, most studies have included younger age groups. Although the general population showed decreasing suicide attempt rates with increasing age, there was no notable change between age groups for those with ASD. The suicide rate was higher among those with ASD who were aged 20 years or older; nevertheless, suicide risk is increasing with age in the general population of Denmark.

A possible causal mechanism linking ASD to suicidality, particularly in adults, may be a combination of social isolation and poor access to health care.^28^ Although it is possible that the inability to establish and retain social and intimate relationships is associated with suicide attempts among adult women with ASD, they might also receive a diagnosis and treatment later in the course of the disorder by being able to camouflage their autistic traits.^29,30^ This possibility might explain the higher rates of suicidal behavior among women in our study, which is supported by findings from Swedish linkage studies in which higher risk of suicidal behavior was noted for female individuals with ASD compared with their controls than for male individuals.^16,31^

The highest rates of suicide attempt were found for people with ASD who were unemployed; however, the difference with the group without ASD was highest for those who were employed, with employment having a lesser protective association for those with ASD. Some authors have suggested a mitigating association of employment for those with ASD.^14^ We can only speculate whether this finding might be partly associated with higher levels of peer victimization and other types of workplace bullying, as shown in other studies.^14^ Another possibility is that those who are employed in low-paying jobs are presumably experiencing stress owing to poor finances and structural inequality.

Increased risk of suicidal behavior among those with psychiatric disorders has been long established for the general population,^10^ and psychiatric comorbidity has also been shown to be associated with increased risk in studies for individuals with ASD.^2,8^ Nevertheless, the association of psychiatric comorbidity with suicidal behaviors of individuals with ASD is still understudied, to our knowledge.^16^ A recent population-based case-cohort study from Sweden found that the risks of suicide attempt and suicide among those with ASD without comorbidities remained significantly higher compared with matched controls after adjusting for comorbidities.^16^ Results from our cohort study showed that individuals with ASD without comorbidities did not have higher rates of suicide attempt or suicide. However, our study might not have had sufficient power or the odds ratios may have overestimated the incidence rate ratios. Our results indicate that psychiatric comorbidity is a major risk factor for suicide attempt and suicide among people with ASD; more than 90% of those with ASD who attempted or died by suicide had another comorbid condition. Anxiety and affective disorders were the most common comorbidities, followed by schizophrenia spectrum disorders and ADHD. Attention-deficit/hyperactivity disorder, the most common comorbid condition among those with ASD, did not increase the rate of suicide attempt compared with individuals who had ASD without comorbid conditions. It has been suggested that suicidality is more common among high-functioning individuals with ASD,^29^ which has been shown in some studies measuring functioning by the presence of intellectual disability.^16,31^ This suggestion is in line with our findings of higher rates of suicide attempt and suicide among those with ASD only (high-functioning group). This result seems to be associated with the finding that, in contrast to the general population, the risk of suicide attempt among people with ASD increased with educational level and was highest among those with a university degree (although a university degree was relatively rare in people with ASD). Higher levels of cognitive functioning and education may imply a wider exposure to different risk factors,^27^ but also there is the realization that limited social and problem-solving skills may increase self-imposed pressure to cope with and alter expectations of success.

### Implications

Our results point toward important implications for clinicians working with people with ASD and those working with suicidal patients, highlighting a need for tailored suicide prevention.^27^ Early intervention to improve social skills in children with ASD is likely to lower risks of suicidal behavior later in life. Nevertheless, it is essential to expand support and services for adults with ASD, especially those with psychiatric comorbidity, considering the higher risk of suicide attempt throughout the life span.^28,32^ The high rates of suicide attempt and suicide among female individuals with ASD suggest a need to improve diagnostic tools to avoid delays in required treatment. Further work to identify the best tools to measure suicidality among those with ASD is needed.^33^

### Strengths and Limitations

This study has some strengths, including the longitudinal nationwide register data with few missing values.^17^ The analyses were adjusted for period effects to avoid an increase in the number of cases over time, which might be associated with different diagnostic criteria. Furthermore, the inclusion criterion of a hospital-based ASD diagnosis, which was determined by a child psychiatrist in a mental health setting using standardized diagnostic tools, was likely to have improved the validity of the measure. In addition to time period, our analyses were adjusted for variations associated with differences with respect to sex and age. Still, the sensitivity analyses supported that significant associations remained when adjusting for potential confounders or restricting the sample to younger adults.

Some limitations should also be noted. Although the diagnosis of childhood autism in the PCRR has been evaluated as valid,^32^ there is less information about possible underreporting of ASD in the registers, and it is possible that we have missed some cases (for instance, if some persons received a diagnosis only in primary care). Suicide attempts are underrecorded in the Danish hospital registries; therefore, our estimates may be considered conservative.^34^

## Conclusions

Our findings show a higher rate of suicide attempt and suicide associated with persons with ASD in a nationwide cohort study in Denmark. Psychiatric comorbidity was found to be a major risk factor, with more than 90% of those with ASD who attempted or died by suicide having another comorbid condition. A number of risk factors for suicidality among individuals with ASD are different from risk factors in the general population.

## Notes

Supplement. **eTable 1.** *ICD* Codes **eTable 2.** Description of People With ASD
