# Supplementary material for: Assessment of Suicidal Behaviors Among Individuals With Autism Spectrum Disorder in Denmark
Source: JAMA Netw Open. 2021 Jan 12;4(1):e2033565. doi: 10.1001/jamanetworkopen.2020.33565 (PMC12578491; doi:10.1001/jamanetworkopen.2020.33565)
Supplement: Supplement. — eTable 1. ICD Codes eTable 2. Description of People With ASD [file jamanetwopen-e2033565-s001.pdf]

Supplementary Online Content

Kõlves K, Fitzgerald C, Nordentoft M, Wood SJ, Erlangsen A. Assessment of suicidal behaviors among individuals with autism spectrum disorder in Denmark. *JAMA Netw Open.* 2021;4(1):e2033565. doi:10.1001/jamanetworkopen.2020.33565

eTable 1. *ICD* Codes
eTable 2. Description of People With ASD

This supplementary material has been provided by the authors to give readers additional information about their work.

© 2021 Kõlves K et al. *JAMA Network Open.*

eTable 1. *ICD* Codes

**Diagnosis**	***ICD-10***	***ICD-8***
Substance use disorders (SUD)	F10-19	291, 294.39, 303, 304
Schizophrenia	F20	295.x9 (excluding 295.79)
Schizophrenia spectrum disorders (SSD)	F20-29	295.x9, 296.89, 297.x9, 298.29, 298.39, 298.89, 299.04, 299.05, 299.09, 301.09, 301.29, 301.83
Affective disorders	F30-39	296.x9, 298.09, 298.19, 300.49, 301.19
Depression	F32-39	296.09, 296.29, 298.09, 300.49
Bipolar disorder	F30-31	296.19, 296.39, 298.19
Anxiety	F40-41, F93	300.09, 300.19, 300.29
Obsessive-compulsive disorder (OCD)	F42	300.39 296.x9, 298.09, 298.19, 300.49, 301.19
Post-traumatic stress disorder (PTSD)	F43.1	NA
Eating disorders	F50	306.50, 306.58, 306.59
Personality disorders	F60	300.19, 301.x9 (excluding 301.19), 301.80, 301.81, 301.82, 301.84
Intellectual disability	F70-79	311.xx, 312.xx, 313.xx, 314.xx, 315.xx
Autism spectrum disorders (ASD)	F84.0, F84.1, F84.5, F84.8, F84.9	299.00-03
Attention deficit hyperactivity disorder (ADHD)	F90, F98.8	308.01
Oppositional defiant disorder/ conduct disorder (ODD/CD)	F91, F92, F90.1	308.03, 308.06

© 2021 Kõlves K et al. *JAMA Network Open.*

eTable 2. Description of People With ASD

Characteristics		N	%
Sex	Male	25,718	73.4
	Female	9,302	26.6
Age groups	10-19	18,779	53.6
	20-29	11,204	32.0
	30-39	2,688	7.7
	40+	2,349	6.7
Education	Basic	22,374	63.9
	Vocational	1,639	4.7
	High school	2,190	6.3
	University degree	1,002	2.9
	Missing/unknown	7,815	22.3
Socioeconomic status	Employed	2,888	8.2
	Unemployed	4,547	13.0
	Disabled/retired	4,988	14.2
	Child/student	20,100	57.4
	Missing/unknown	2,497	7.1
Civil status	Married/registered partnership/cohabiting	14,859	42.4
	Not married/registered partnership/cohabiting	11,950	34.1
	Missing/unknown	8,211	23.4
Parental psychiatric disorders	No	24,477	69.9
	Yes	10,543	30.1
Parental suicidal behaviour	No	32,953	94.1
	Yes	2,067	5.9
Charlson Comorbidity Index	None	30,891	88.2
	1+	4,129	11.8
Psychiatric comorbidity	Any other psychiatric disorder	25,401	72.5
	Substance use disorder	1,206	3.4
	Schizophrenia[a]	1,578	4.5
	Schizophrenia spectrum disorders	3,833	10.9
	Affective disorders	5,770	16.5
	Depression[b]	5,275	15.1
	Bipolar disorder[b]	477	1.4
	ADSO	9,646	27.5
	Anxiety[c]	3,525	10.1
	Obsessive compulsive disorder (OCD)[c]	1,955	5.6
	Post-traumatic stress disorder (PTSD)[c]	181	0.5
	Eating disorders	835	2.4
	Personality disorders	3,314	9.5
	Borderline personality disorder	395	1.1
	ADHD	11,456	32.7
	Oppositional defiant disorder/ Conduct disorder (ODD/CD)[d]	2,217	6.3

© 2021 Kõlves K et al. *JAMA Network Open.*

	Intellectual disability	5,296	15.1
Age at first ASD diagnosis	<13 years	19,486	55.6
	13-24 years	12,259	35.0
	≥25years	3,275	9.4
Period[e]	1995-1999	22	0.1
	2000-2004	64	0.2
	2005-2009	103	0.3
	2010-2016	34,831	99.5

[a] Subgroup of schizophrenia spectrum disorders

[b] Subgroup of affective disorders

[c] Subgroup of anxiety, dissociative, stress-related, somatoform and other nonpsychotic mental disorders (ADSO)

[d] Subgroup of neurodevelopmental disorders

[e] Period: the number of people with ASD who were censored

© 2021 Kõlves K et al. *JAMA Network Open.*
